# A European cross-sectional survey to investigate how involved doctors training in clinical pharmacology are in drug concentration monitoring

**DOI:** 10.1007/s00228-022-03316-z

**Published:** 2022-04-14

**Authors:** Thomas J. Green, Lauren E. Walker, Richard M. Turner

**Affiliations:** 1 Liverpool University Hospitals NHS Foundation Trust, Liverpool, UK; 2 The Wolfson Centre for Personalised Medicine, University of Liverpool, Liverpool, UK

**Keywords:** Therapeutic drug monitoring, Clinical pharmacology, Training, Survey

## Abstract

**Purpose:**

Therapeutic drug monitoring (TDM) is widely recognised as a key attribute of clinical pharmacologists; yet, the extent to which physicians undertaking postgraduate training in clinical pharmacology (hereafter trainees) are involved in TDM is poorly characterised. Our own experience suggests wide variation in trainee exposure to TDM.

**Method:**

We performed a Europe-wide cross-sectional internet-based survey of trainees to determine the nature and extent of trainee involvement in TDM.

**Results:**

There were 43 responses from eight countries analysed. Of the 21 respondents from the UK, all were also training in general internal medicine (GIM), while all of the respondents who were solely training in clinical pharmacology were from outside the UK. Overall, 86.0% of respondents reported access to drug monitoring for clinical care at their affiliated institution, of which 81.0% were personally involved in TDM in some capacity. On average, trainees reported that drug monitoring was available for 16 of the 33 (48%) of the drug/drug classes surveyed. UK-based respondents were involved in requesting drug-level investigations and interpreting the results for patients under their care in 76.2% and 85.7% of cases, respectively, while non-UK respondents supported other healthcare professionals to interpret results in 45.4% of cases. Trainees felt TDM training was generally either insufficient or very inadequate.

**Conclusion:**

While access to TDM is relatively available at institutions where trainees are based, the role of trainees is variable and affected by a variety of factors including country and training programme. Universally, trainees feel they need more education in TDM.

## Introduction

In 2012, the World Health Organisation (WHO), in partnership with the International Union of Basic and Clinical Pharmacology (IUPHAR) and Council for International Organizations of Medical Sciences (CIOMS), published a position paper on the role of clinical pharmacologists in relation to healthcare, teaching and research [1]. Within this report, they listed therapeutic drug monitoring (TDM) as one of the key clinical services of clinical pharmacology to aid patient care. TDM is the measurement of the concentration of a drug/drug metabolite in blood plasma/serum to guide clinical care. While determination of drug concentrations is central to research studies investigating a drug’s pharmacokinetics, TDM is important in clinical care to guide dose-making decisions and help investigate clinical presentations of altered drug efficacy or adverse reactions, particularly for drugs with a narrow therapeutic window (e.g. tacrolimus). Although not strictly TDM, screening to detect the presence or absence of a drug (e.g. antihypertensive drugs) in blood or urine is also important when investigating drug adherence in clinical practice. Importantly, a comprehensive TDM service should include clinical interpretation of assay results, which involves consideration and integration of other relevant patient-specific factors (e.g. age, comedications, renal function, pharmacogenomics) [1]; thus, the clinical and pharmacological training of physician clinical pharmacologists should ideally position them to offer a full TDM service.

Nevertheless, clinical pharmacology is a diverse specialty and the spectrum and extent of its activities vary between countries. In 2013, a survey was undertaken of senior national delegates on the Council of the European Association for Clinical Pharmacology & Therapeutics (EACPT) on the development of clinical pharmacology in Europe with particular regard to healthcare. Interestingly, this survey found that clinical pharmacologists were responsible for TDM in just 16 of 31 European countries [2].

The extent and types of involvement of clinical pharmacologists in TDM are difficult to ascertain. Although the WHO position paper recommends that TDM services are ideally provided by clinical pharmacology, it also acknowledges that the form of this and other clinical pharmacological services will depend upon the resources available and will likely vary between facilities and countries [1]. Moreover, reports from clinical pharmacologists within the literature are sparse, often stating that clinical pharmacology has a role in TDM, but offering few further details [3, 4]. Importantly, little is known about the variability and extent of training in and exposure to TDM during postgraduate clinical pharmacology medical training programmes across Europe. However, in our own anecdotal experience from discussions with clinical pharmacology postgraduate physician colleagues at international meetings, there appears wide variation in involvement and exposure to TDM while training in clinical pharmacology.

Therefore, the primary aim of this study was to determine the extent and nature of involvement of physicians training in clinical pharmacology in TDM and drug detection screening through a survey. Additionally, it was intended that the survey results might cautiously be used to estimate, by proxy, the availability of drug concentration testing at sites where clinical pharmacologists are being currently trained in different European counties.

## Method

### Study design

A pilot cross-sectional survey intended for medically qualified doctors undergoing postgraduate training in clinical pharmacology (hereafter trainees) in Europe was performed, utilising the online questionnaire provider, SurveyMonkey®. The survey was distributed via e-mail. This e-mail was sent to both individuals that had previously registered for the young clinical pharmacologist’s pre-meeting of the 2019 EACPT Congress through the EACPT Working Group of Young Clinical Pharmacologists, and specialty registrar doctors in Clinical Pharmacology & Therapeutics registered with the British Pharmacological Society (BPS). The invitation e-mail contained a description of this project, a link to the survey and a request to forward the survey to other local clinical pharmacology trainees whom the recipient was aware of, with the proviso of informing the survey research team of the number and country location of any additional recipients, to enable accurate tallying of the number invited to partake in the survey. The e-mail was initially sent on 01/Jul/2020 with two subsequent follow-up reminders. The survey was open for completion between 01/Jul/2020 and 30/Sept/2020. Ethical approval to undertake this research was obtained from The University of Liverpool Health and Life Sciences Research Ethics Committee (UK). All responses were anonymous and the preset SurveyMonkey® function to record the IP address of respondents was deselected to maintain anonymity.

### Questionnaire

The questionnaire was written in English. Prior to completing the survey, potential participants were presented with the aims of the project and asked to consent to their participation in the study. The survey consisted of 28 questions, of which six were automatically skipped if deemed no longer relevant based on the participant’s prior answers. The main sections of the survey were as follows: respondent demographics, training and qualifications, involvement in and availability of TDM and drug screening, and their opinions on training and confidence with interpreting results. In the survey, drug screening was defined as leading to a qualitative clinical report (for instance, stating that a drug has or has not been detected), while TDM would lead to a quantitative clinical report (concentration reported).

### Analysis

Anonymous responses were automatically tabulated by the survey website, with results downloaded into Microsoft Excel for further analysis and interpretation. All survey respondents were included in the analyses, except for those that did not complete the survey, and respondents who were not both a medically qualified doctor and postgraduate trainee in clinical pharmacology according to the demographics section of their survey response.

## Results

### Demographics

The survey was distributed to 169 potential respondents. Of these, 69 (40.8%) responded and 61 (36.1%) completed the survey in full. Three (1.8%) were excluded as they were based outside Europe, eight (4.7%) were consultant grade or equivalent and seven (4.1%) were non-medical trainees (e.g. pharmacy graduates). Therefore, 43 (25.4%) respondents were a qualified medical doctor, a postgraduate trainee (e.g. resident or registrar) in clinical pharmacology, based in Europe, and completed the survey; the primary results presented here are based on this cohort.

The demographics of this cohort are in Table 1. The UK represented 21 (48.8%) of these 43 responses, with all UK trainees also training in general internal medicine (GIM); all of those training solely in clinical pharmacology (*n* = 19, 44.2%) were from continental Europe. Ten (23.3%) trainees also held PhDs in addition to their primary medical qualification. The majority (79.0%) of respondents were working in a public hospital and had links to a university.

**Table 1:** Demographics of respondents

**Characteristic**	***n***	**%**
*Base country*		
UK	21	48.8
Sweden	7	16.3
Spain	6	14.0
Portugal	3	7.0
Lithuania	2	4.7
Estonia	2	4.7
Ireland	1	2.3
Netherlands	1	2.3
*Qualification*		
Medical degree	33	76.7
Medical degree + PhD	10	23.3
*Years qualified as a medical doctor*		
1 to 2	2	4.7
3 to 5	13	30.2
5 to 10	22	51.2
> 11	6	14.0
*Years training in Clinical Pharmacology and Therapeutics*		
1	7	16.3
2	11	25.6
3	3	7.0
4	8	18.6
5	7	16.3
6 to 9	6	14.0
At least 10	1	2.3
*Training/trained in other speciality*		
General Internal Medicine	21	48.8
Cardiology	1	2.3
Psychiatry	1	2.3
Dermatology	1	2.3
Paediatrics	1	2.3
None	18	41.9
*Primary institution*		
Public hospital	37	86.0
University	6	14.0
*Secondary institution*		
Public hospital	6	14.0
University	28	65.1
Drug regulator	1	2.3
None	8	18.6

### Availability of services

Thirty-seven respondents (86.0%) indicated that TDM for clinical care is available at their affiliated institution. The breakdown of these 37 affirmative responses by country was as follows: 18 (85.7%) for UK-based respondents, five (71.4%) in Sweden, five (83.3%) in Spain, three (100%) in Portugal, two (100%) in Estonia, two (100%) in Lithuania, one (100%) in Ireland, and one (100%) in the Netherlands. Of the six (14.0%) individuals who reported that their institution did not measure and report drug concentrations for clinical care, five were based at public hospitals and one at a university affiliated to a hospital.

When asked who provides the service for measuring drug concentrations, respondents answered clinical chemistry/biochemistry in 24 (64.8%) of cases, pharmacy in seven (18.9%) of cases, clinical pharmacology in one (2.7%) case, and five (13.5%) stated a combination of these service providers. Interestingly, all respondents from the UK, Sweden, Ireland and Lithuania answered clinical chemistry/biochemistry, while all respondents from Portugal, the Netherlands and all but one from Spain (5 of 6) answered pharmacy. Two of the 21 UK respondents reported that some services were outsourced or samples were sent out of their institution.

**Fig. 1:**
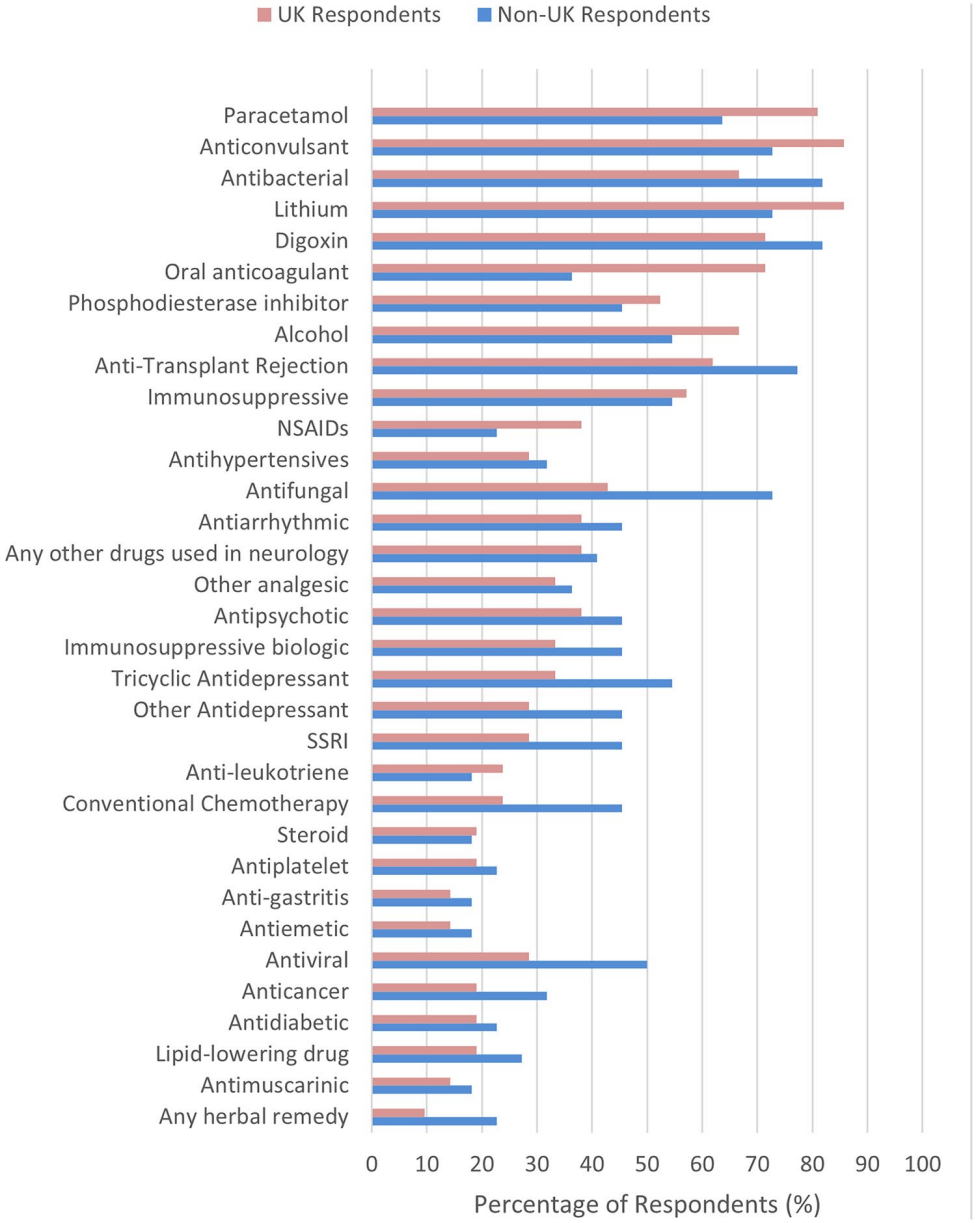
Availability of TDM by drug/drug class reported by respondents. Other antidepressant: not an SSRI or TCA (e.g. SNRI). Immunosuppressive: not an anti-transplant rejection, steroid or biologic immunosuppressive. Other analgesic: not an NSAID or paracetamol

**Fig. 2:**
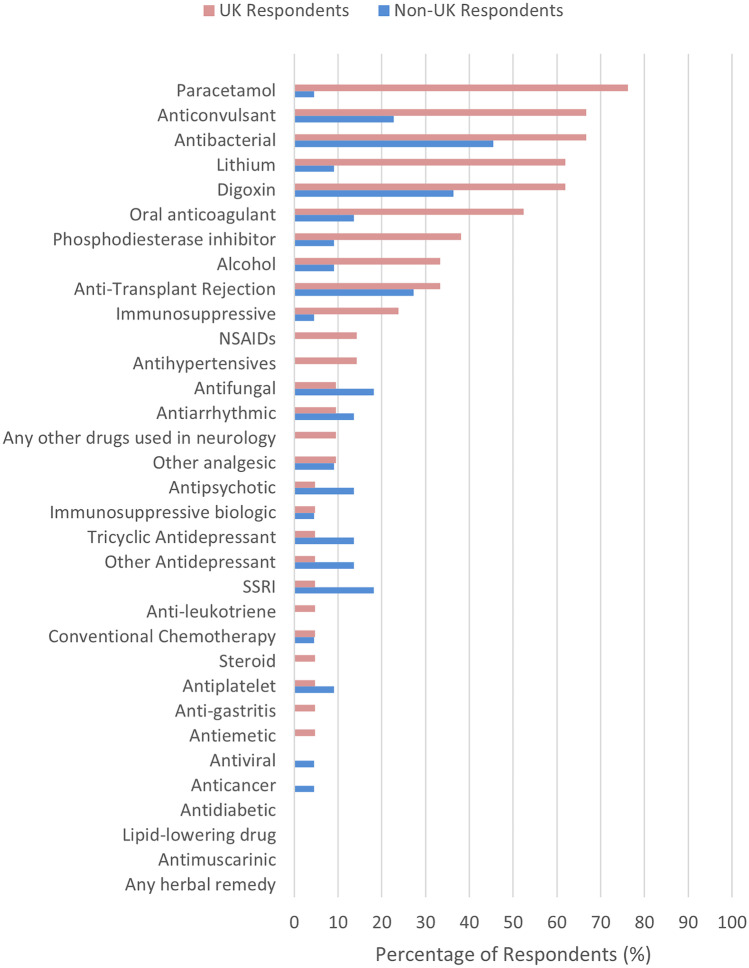
Personal involvement of respondents in TDM by drug/drug class. Other antidepressant: not an SSRI or TCA (e.g. SNRI). Immunosuppressive: not an anti-transplant rejection, steroid or biologic immunosuppressive. Other analgesic: not an NSAID or paracetamol

A breakdown of drug monitoring availability and respondent involvement, by drug class, is reported in Figs. 1 and 2. As can be seen, the major drugs/drug classes where over 50% of respondents indicated that drug monitoring was available at their institute were for anti-bacterial drugs, anti-fungal drugs, digoxin, lithium, paracetamol, alcohol, anti-convulsant drugs, phosphodiesterase inhibitors, oral anticoagulants, anti-transplant rejection drugs and other (non-biologic) immunosuppressants. Overall, we asked if 33 different drugs or drug classes were specifically available for measurement at the respondent’s institution. After grouping respondents by country, the average number of these drugs or drug classes considered available for measurement at their institute were as follows: 15.2 (46.1%) in the UK, 20.4 (61.8%) in Sweden, 8.6 (26.1%) in Spain, 17.3 (52.5%) in Portugal, 19.0 (57.6%) in Lithuania, 16.5 (50.0%) in Estonia, 16.0 (46.1) in the Netherlands and 33.0 (100%) in Ireland.

A specific comparison of both availability of TDM and respondent involvement in TDM (in any capacity), by drug/drug class, between UK and non-UK respondents is shown in Figs. 1 and 2. Availability was broadly similar between UK and non-UK respondents, although there appeared greater nominal TDM availability for antiviral drugs, antifungal drugs, conventional chemotherapy drugs, tricyclic antidepressants, SSRIs, and other antidepressants in the non-UK group. On the other hand, UK respondents reported more involvement in the monitoring of paracetamol, lithium, anticonvulsants and oral anticoagulants.

When asked if patients at their institution could undergo an anti-hypertensive drug adherence screen, 17 (39.5%) responded yes, 14 (32.6%) no and 12 (27.9%) were unsure. Of the UK respondents, 16/21 (76.2%) were able to screen for adherence, while all of the respondents from Sweden, Spain, Portugal, the Netherlands and Lithuania answered either no or unsure. Screening for drugs of abuse was available to 36 (83.7%) of respondents, while seven (16.3%) reported either no or they were unsure about availability of drugs of abuse screening at their institute. When asked if a respondent’s institution provided screening for any other drug, drug class or anti-drug antibodies, five (11.6%) responded no, 33 (76.7%) were unsure, while five (11.6%) responded affirmatively; examples given included anti-TNF antibodies and erythropoietin.

Respondents were asked if there were any drugs that screening for, or measuring the concentrations of, would improve patients care in their opinion, but are not yet available at their institution. Thirty-one (72.1%) replied that they did not know, while five (11.6%) responded no and seven (16.3%) yes; when the latter were asked which drugs or classes should be assayed, responses included anti-microbials, immunosuppressants, antidepressants, antiarrhythmics, antihypertensives and anticonvulsants.

### Role of trainees in drug concentration monitoring

Of the 37 (86.0%) respondents with TDM available for clinical care at their affiliated institution, 30 (81.0%) were involved in drug monitoring in some form. There was at least one respondent in each represented country that reported involvement in TDM. Of all respondents, 21 (48.8%) request drug concentration tests for patients, 15 (34.9%) help other healthcare professionals to interpret results and/or 21 (48.8%) use drug concentration results to manage patients under their own care. No respondents were involved in assisting the laboratory service to assay the drug concentrations. All the respondents from Estonia and Lithuania, and five (71.4%) from Sweden assisted others with interpretation of results. However, in the UK, 16 (76.2%) respondents reported requesting tests for patients and 18 (85.7%) use drug monitoring to manage patients under their own care. All but one respondent from Spain reported no involvement in drug concentration monitoring.

When asked how often respondents have been personally involved in TDM over the last 12 months, zero (0.0%) reported daily, 14 (32.6%) weekly, six (14.0%) monthly, nine (20.9%) every 3 months and eight (18.6%) none for the last 12 months. Weekly involvement in drug concentration monitoring based on the respondent’s duration of training in clinical pharmacology was three (60.0%), six (66.7%), zero (0.0%), two (25%), two (40%), one (20%) and zero (0.0%) for 1 year, 2 years, 3 years, 4 years, 5 years, 6–9 years and > 10 years respectively. A breakdown by country of respondent can be seen in Table 2. Overall, more of the UK respondents appeared personally involved in TDM (18/21, 85.7%) than their non-UK colleagues (12/22, 54.5%) (Table 2, Figs. 1 and 2).

**Table 2:** Frequency of respondents’ involvement in drug screening and drug monitoring by base country

Base country of respondent	*n*	Involvement in drug screening (%)						Involvement in drug monitoring (%)					
		Daily	Weekly	Monthly	Every 3 months	Once	Never	Daily	Weekly	Monthly	Every 3 months	Once	Never
UK	21	-	14.3	28.6	23.8	14.3	19.0	-	33.0	23.8	28.6	-	14.3
Non-UK	22	-	13.6	13.6	27.3	4.5	40.9	-	36.8	5.3	15.8	-	42.1
* Sweden*	*7*	-	-	28.6	14.3	-	57.1	-	42.8	-	-	-	57.1
* Spain*	*6*	-	16.7	-	16.7	16.7	50.0	-	16.7	-	16.7	-	66.7
* Portugal*	*3*	-	-	-	66.7	-	33.3	-	-	-	33.3	-	66.7
* Estonia*	*2*	-	50.0	-	50.0	-	-	-	100.0	-	-	-	-
* Lithuania*	*2*	-	50.0	50.0	-	-	-	-	50.0	50.0	-	-	-
* Ireland*	*1*	-	-	-	100.0	-	-	-	-	-	100.0	-	-
* Netherlands*	*1*	-	-	-	-	-	100.0	-	-	-	-	-	100.0

### Role of trainees in drug screening

When asked about personal involvement with drug screening (e.g. anti-hypertensive drug adherence detection) within the last 12 months, no trainees (0.0%) reported daily involvement, six (13.9%) weekly, nine (20.9%) monthly, 11 (25.6%) every 3 months and four (9.3%) once. Thirteen (30.2%) trainees reported no involvement with drug screening. In the UK, 17 (81.0%) reported at least some personal involvement with drug screening within the last 12 months, whereas four (19.0%) reported none. Of the non-UK-based respondents, 13 (59.1%) reported at least some involvement whereas nine (40.9%) reported none (Table 2).

When asked about their specific involvement in drug screening, 23 (53.5%) request the test for patients, 22 (51.2%) use the results in the management of their patients and/or 13 (30.2%) assist other doctors or healthcare workers with the interpretation of results.

### Provision of training

**Fig. 3:**
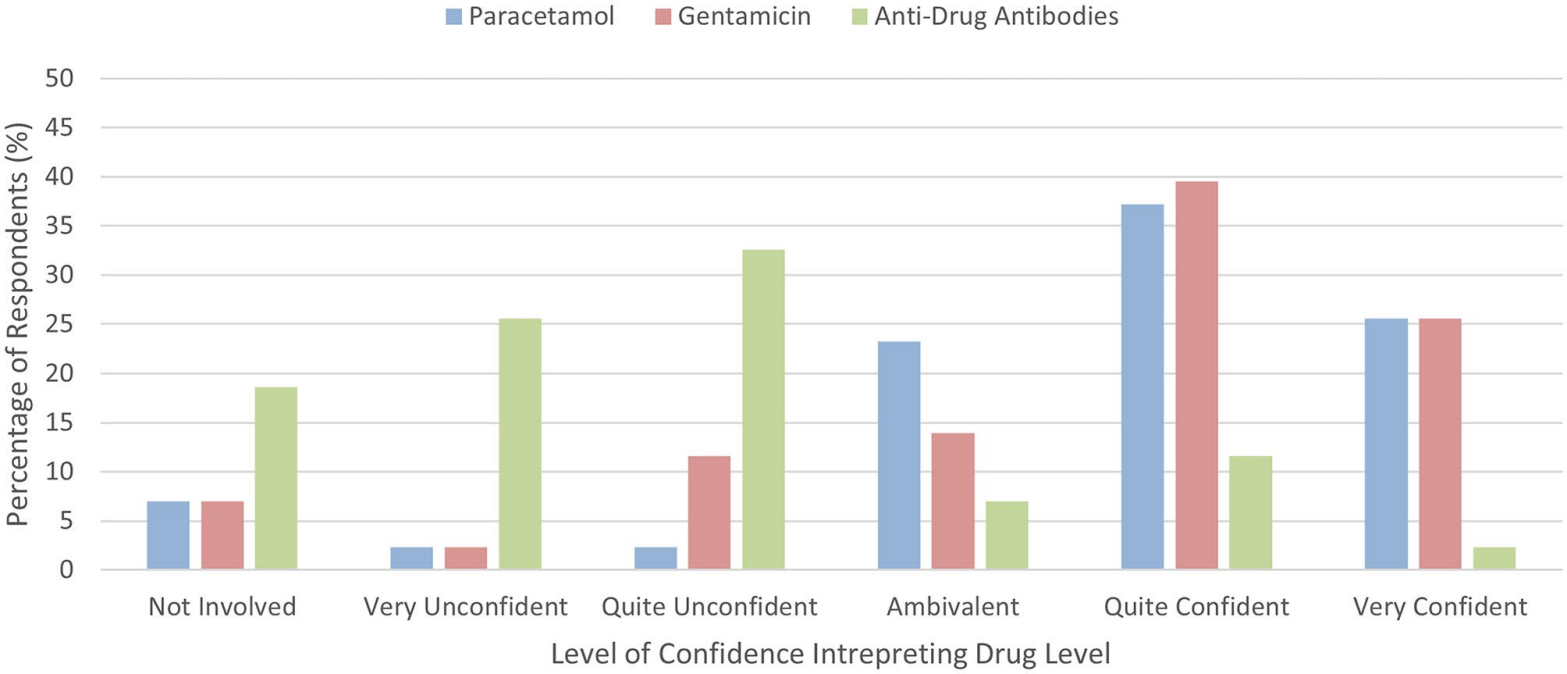
Confidence with interpretation of drug concentrations

Respondents were asked about the amount of training they receive on drug monitoring and screening within their clinical pharmacology curriculum. No respondents indicated that they receive too much training, while nine (20.9%) reported it being just right, 25 (58.1%) insufficient, eight (18.6%) very inadequate and one answered that they did not know (2.3%). Breaking this down by country, respondents answered insufficient 10 (47.6%), five (71.4%), five (83.3%) and one (33.3%) times for the UK, Sweden, Spain and Portugal, respectively. For the answer very inadequate, the number of respondents was four (19.0%), one (14.3%), one (16.7%) and two (66.7%) for the UK, Sweden, Spain and Portugal, respectively. Figure 3 shows respondents’ confidence with interpreting drug concentration results for paracetamol, gentamicin and anti-drug antibodies. With regards to interpretation of gentamicin concentrations, 65.1% of respondents answered quite confident or very confident, while 62.8% gave these answers for paracetamol concentration interpretation. Only 13.9% of respondents felt quite confident or very confident with interpretation of anti-drug antibody titres.

When asked which specific areas they would like further training in, 38 (86.4%) indicated the evidence base for monitoring/screening specific drugs, 22 (51.2%) the laboratory processes for monitoring/screening drugs and 38 (88.4%) with interpreting results. No respondents felt they did not need further training in drug monitoring. Respondents felts barriers to further training included a lack of time (51.2%), financial pressures (20.1%), lack of evidence for drug monitoring (6.9%), lack of evidence for drug screening (2.3%), services being run by another specialty (51.2%), services being run by another institution (9.3%) and 18.6% felt it would not be permissible within the current training curriculum. Free text responses highlighted difficulties in identifying someone with the time to teach, a lack of clarity around specific learning objectives and expectations of trainees, and a failure of specialists to acknowledge the learning requirements of trainees. Five (11.6%) respondents felt there would be no barriers to further training.

## Discussion

Our survey has explored and provided a detailed insight into the role of clinical pharmacology physician postgraduate trainees across Europe in TDM, which, to our knowledge, is the first of its kind. The main findings were as follows: (1) in centres where clinical pharmacology trainees are based, drug monitoring is often available, albeit with large variation in which drugs can be monitored; (2) individual involvement in drug monitoring was reasonably high but with variation in the type of involvement; (3) all UK trainees dual train in clinical pharmacology and GIM, whereas the non-UK trainees that responded to this survey tended to not dual specialise; (4) UK trainees are thus more likely to request investigations and use drug monitoring to manage patients under their own care, while non-UK trainees are more likely to assist others with interpretation of results, and; (5) training in drug monitoring was felt to be insufficient or very inadequate by the majority of trainees regardless of country of working.

TDM was identified as a key role of clinical pharmacologists in patient care; yet in a survey of 31 European countries, only 16 reported involvement in TDM [1, 2]. This previous survey did not detail which countries clinical pharmacologists were, or were not, involved in TDM. In our survey, overall involvement in TDM was high, with 86% having access to TDM and, of these, 81% reported personal involvement. Seven of the eight countries had at least 50% of the respondents involved in TDM, with Spain being the only exception (16.7%). A free text response from a respondent based in Spain reported TDM being the responsibility of the pharmacy and clinical biochemistry departments.

In the UK, 18 of the 21 respondents were personally involved in TDM. The situation for the three respondents without involvement is unclear, as they all worked in public hospitals affiliated with universities. There are no data in the literature for comparison and the UK National Trainee Survey does not ask trainees for their involvement in TDM [5]. The role of UK trainees differs from that of their Continental European counterparts with involvement primarily in requesting tests and using TDM to manage patients under their own care, rather than assisting others with interpretation of results. As all UK respondents were also training in GIM, it is unclear which aspect of their training the TDM role falls under.

Respondents to our survey felt their training was lacking in monitoring/screening of drugs, with 76.7% describing it as “insufficient” or “very inadequate”. Specific areas of training need were: understanding the evidence base for monitoring/screening of specific drugs, and interpreting results. Curriculums are sparse in specifics regarding intended learning outcomes for TDM, which was a fact commented on by some respondents. Nevertheless, this lack of training was not reflected in the trainees’ confidence in interpreting drug concentrations for the commonly prescribed medications, paracetamol and gentamicin (Fig. 3).

Training in clinical pharmacology occurs through experiential learning, peer teaching and formal postgraduate teaching [6]. Training in TDM should thus encompass both theoretical and experiential learning. It is important for clinical pharmacology trainees to understand relevant pharmacokinetic principles, characteristics of a drug that make it suitable for TDM, and clinical indications for TDM. Experiential learning is necessary to gain practical competence in applying TDM (e.g. optimal post-dose sampling time), interpreting results and making appropriate clinical decisions that integrate TDM with a patient’s full clinical picture. Importantly though, respondents felt a lack of time was the most significant barrier to training, along with comments on difficulties finding an expert with the time to teach. It has previously been noted that clinical pharmacology has difficulties delivering comprehensive training and peer teaching given the small nature of the specialty and limited numbers of staff at each site [6]. Therefore, TDM eLearning modules designed specifically for clinical pharmacology trainees could be designed, similar to those available to pharmacists, as flexible eLearning can fit around clinical workloads [7]. Specific clinical pharmacology training sessions delivered virtually should focus on TDM with multiple real-world case examples. The development of virtual national monthly training sessions for UK-based clinical pharmacology trainees was driven by the COVID-19 pandemic, but virtual-based teaching offers an efficient mechanism for a small number of TDM experts to reach a wide (e.g. national) clinical pharmacology trainee audience [8]. Initiatives such as this should be expanded to other European countries that do not yet have a national virtual teaching programme. In addition, TDM-focused joint training with clinical pharmacists and clinical biochemists should be encouraged, in both formal teaching sessions and experiential ward or laboratory-based settings.

The results of this survey should be viewed in the context of the following limitations. Firstly, some of the countries had small numbers of respondents (Ireland, the Netherlands, Estonia and Lithuania), while we received no respondents from other European countries. Secondly, almost half the responses were from UK based trainees. Therefore, a follow-up initiative to obtain further data from a range of European countries would be useful. Thirdly, the reported availability of services at institutions is based on respondents’ awareness of them and may not fully reflect the actual services available. Fourthly, the survey was sent in English, and while this may have impacted the response rate, it was considered that most non-UK-based clinical pharmacologists involved in the EACPT were likely to have a reasonable proficiency in written English. Finally, responses on training needs were subjective with expectations and standards likely to vary between respondents, which may not reflect the competence of a trainee.

In summary, this project has surveyed the extent of involvement, availability and training in drug monitoring and screening of clinical pharmacology trainees from different European countries. While TDM is generally available, trainees have a range of involvement and feel they lack appropriate training. Addressing the educational needs around TDM amongst clinical pharmacology trainees should be a priority if they are to become competent leaders and service providers in this area.
